# Neurobiological Foundations of Acupuncture: The Relevance and Future Prospect Based on Neuroimaging Evidence

**DOI:** 10.1155/2013/812568

**Published:** 2013-05-14

**Authors:** Lijun Bai, Lixing Lao

**Affiliations:** ^1^The Key Laboratory of Biomedical Information Engineering, Ministry of Education, Department of Biomedical Engineering, School of Life Science and Technology, Xi'an Jiaotong University, Xi'an 710049, China; ^2^Center for Integrative Medicine, School of Medicine, University of Maryland, 520 W. Lombard Street, Baltimore, MD 21201, USA

## Abstract

Acupuncture is currently gaining popularity as an important modality of alternative and complementary medicine in the western world. Modern neuroimaging techniques such as functional magnetic resonance imaging, positron emission tomography, and magnetoencephalography open a window into the neurobiological foundations of acupuncture. In this review, we have summarized evidence derived from neuroimaging studies and tried to elucidate both neurophysiological correlates and key experimental factors involving acupuncture. Converging evidence focusing on acute effects of acupuncture has revealed significant modulatory activities at widespread cerebrocerebellar brain regions. Given the delayed effect of acupuncture, block-designed analysis may produce bias, and acupuncture shared a common feature that identified voxels that coded the temporal dimension for which multiple levels of their dynamic activities in concert cause the processing of acupuncture. Expectation in acupuncture treatment has a physiological effect on the brain network, which may be heterogeneous from acupuncture mechanism. “Deqi” response, bearing clinical relevance and association with distinct nerve fibers, has the specific neurophysiology foundation reflected by neural responses to acupuncture stimuli. The type of sham treatment chosen is dependent on the research question asked and the type of acupuncture treatment to be tested. Due to the complexities of the therapeutic mechanisms of acupuncture, using multiple controls is an optimal choice.

## 1. Introduction

Acupuncture is an ancient East Asian healing modality that has been in use for more than 2000 years. Together with herbal medicine, it is regarded as one of the two most pivotal medical skills in East Asian medicines. In the last decades, acupuncture has gained great popularity as an alternative and complementary therapeutic intervention in the western medicine [1]. An estimation of 3 million American adults receive acupuncture treatment each year [2]. Acupuncture is the insertion and stimulation of needles at specific acupoints on the body to facilitate recovery of health. For example, a promising efficacy of acupuncture has been shown in the treatments of postoperative and chemotherapy nausea and vomiting [3]. It has also become a beneficial adjunct for pain management [4, 5]. In spite of its public acceptance, increasing attentions are paid to explore the scientific explanation regarding the physiological mechanism of acupuncture. 

Abundant evidence from animal studies has demonstrated that acupuncture stimulation can facilitate the release of certain neuropeptides in the central nervous system (CNS), eliciting profound physiological effects and even activating self-healing mechanisms [6, 7]. Studies of electroacupuncture in rats revealed that both low-frequency and high-frequency stimulation could induce analgesia, but that there are differential effects of low- and high-frequency acupuncture on the types of endorphins released [8]. One recent study demonstrated that peripheral acupuncture stimulation can ease pain by triggering a natural painkilling chemical called adenosine, which typically surges in concentration after any stress or injury. Adenosine works by docking at a protein called the adenosine A1 receptor, which has well-established roles in suppressing pain and is found on neurons that transmit pain signals [9]. Although animal research clearly supports a role for specific neural pathways underlying the action of acupuncture, it is difficult to interpret these studies in the context of more complex human experience, including the belief states, emotion, and cognition changes. The noninvasive functional magnetic resonance imaging (fMRI) technique has opened a “window” into the brain, allowing us to investigate the central physiological functions involved in acupuncture administration of human beings available. The wide range of physical effects exerted by acupuncture and its purported efficacy for a compendium of clinical pathologies suggest that the brain may be responsible for transmitting the needle stimulus into signals aimed at maintaining homeostatic balance within and across functional subsystems [10–12]. In this review, we have systematically researched and reviewed the literature looking at the neurophysiologic mechanisms of acupuncture on human brain and discussed how these findings contribute to current hypotheses of acupuncture action.

## 2. Neural Correlates Involving Acupuncture in Human

Converging evidence from fMRI studies on acupuncture at commonly used acupoints have revealed significant modulatory effects at widespread cerebrocerebellar brain regions. These regions process information in circuits that can broadly be assumed to engage endogenous antinociceptive limbic networks as well as higher-order cognitive and affective control centers within the prefrontal cortex and medial temporal lobe [13–20]. Researches from Wu et al. indicated that stimulation at LI4 and ST36 resulted in increases in signal intensity of the hypothalamus and nucleus accumbens, as well as decreases in the rostral part of the anterior cingulated cortex, amygdala, and hippocampus [18]. This evidence supported the hypothesis that acupuncture can activate the structures of descending antinociceptive pathway and deactivate multiple limbic areas subserving affective dimension of pain, largely overlapping with the “neuromatrix” for both pain transmission and perception. These regions process information in circuits that can broadly be assumed to engage: the affective (amygdala, hippocampus), sensory (thalamus, primary (SI) and secondary (SII) somatosensory cortices), cognitive (ACC, anterior insula), and inhibitory (PAG, hypothalamus) processing during the experience of pain. Notably, Hui et al. reported that needle stimulation at ST36 induced a wider range of negative signal changes in the limbic-cerebellum system [14]. This widely decreased limbic-cerebellum network may be one of the central characteristics involved in the action of acupuncture. Collectively, neuroimaging data strongly suggest that acupuncture modulates many distributed cortical and subcortical (i.e., brainstem, limbic, and cerebellum) brain areas. These brain areas may contribute to the therapeutic effect of acupuncture by shifting autonomic nervous system (ANS) balance and altering the affective and cognitive dimensions of pain processing.

Previous neuroimaging studies on acupuncture focus mainly on the spatial distribution of brain activities induced by acupuncture stimuli. Interest in exploring what happens in the human brain when subjects do not perform cognitively demanding tasks has increased in the past few years. Some researchers indicated that even in the task-free state, the brain continuously expends a considerable amount of energy, and external tasks only modestly modulate the effects of such ongoing activity [21–23]. Therefore, as suggested by Raichle and colleagues, in terms of overall brain functions, the ongoing intrinsic activity within various brain systems may be at least as important as the activity evoked by external stimuli [22, 23]. More importantly, a recent study has reported differences in resting-state brain functions of people with chronic pain in contrast with controls, and the authors proposed that this difference in resting-state brain activity might reflect the cognitive and affective complications of chronic pain [24]. Therefore, analysis of resting-state connectivity can not only help us to better understand the long-term effects of pain on brain but also the potential benefits of acupuncture in pain treatments. One pioneer studies using independent component analysis have found that acupuncture, not the sham condition, can enhance interregional functional connectivity within both the default mode network and sensorimotor network [25], including the medial temporal lobe, PAG, and supplementary motor area (SMA). They also reported that connectivity between the hippocampus and DMN saliently correlated with parasympathetic output only following the acupuncture stimulation. This indicated that acupuncture may operate through the regulation of autonomic nervous system, which was consistent with increasing evidence for the involvement of autonomic efferent nerve activity underlying its specific effects [26, 27]. Another study using the spontaneous activity detection approach indicated that acupuncture may not only enhance the dichotomy of the anticorrelated resting networks (“default mode” network and “central-executive” network) but also modulate a larger spatio-temporal extent of spontaneous activities in the salient interoceptive-autonomic network, which may contribute to potential actions in the endogenous pain-modulation circuits and homeostatic control mechanism [28]. This hypothesis needs further investigations in the altered and/or dysfunctional brain networks such as those in patients with chronic pain.

## 3. Time-Varied Acupuncture Effect and Its Influence on fMRI Study 

**Figure 1:**
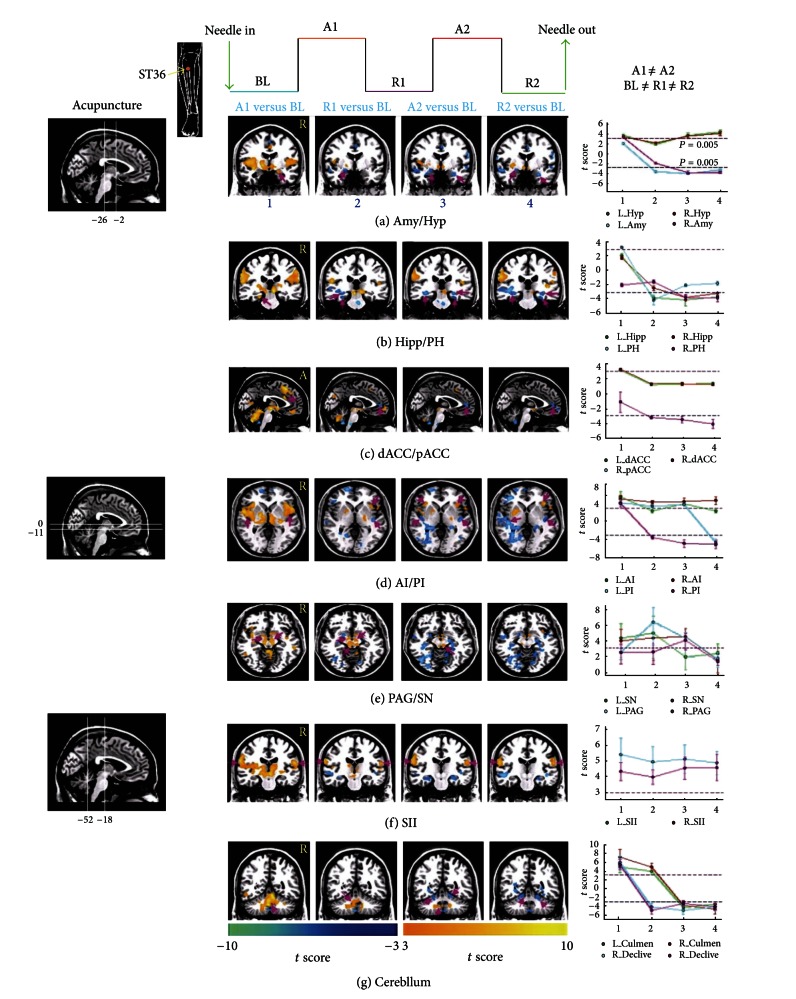
Representative brain areas induced by acupuncture at ST36 for different epochs of multiblock design paradigm (*P* < 0.005, uncorrected). The kinetics of acupuncture was complex and longer acting as a function of time, rather than conforming to simple “on-off” variations predicated by the block-based GLM analysis. Abbreviations: Amy: amygdala; Hipp: hippocampus; PH: parahippocampus; pACC: pregenual anterior cingulate cortex; dACC: dorsal cingulate cortex; AI: anterior insula; PI: posterior insula; SII: secondary somatosensory cortex; Hyp: hypothalamus; PAG: periaqueductal gray; SN: substantia nigra (adapted from [31]).

Abundant clinical reports have indicated that acupuncture can provide relief even beyond the time it is being performed. Psychophysical analysis from Price et al. suggests that the analgesic effects of acupuncture might actually peak long even after the needling session is terminated [29]. Another evidence using pain threshold to potassium iontophoresis also showed the delayed development of acupuncture analgesia [30]. The increase of the pain threshold induced by acupuncture has a peak occurring 20–40 min after needle insertion, and the pain threshold persisted over 30 min after withdrawal of the acupuncture needle. Due to the sustained effect of acupuncture, the temporal aspects of the BOLD response to acupuncture may violate the assumptions of the block-designed GLM estimates (Figure 1) [31]. In the framework of GLM, a specific stimulus sequence (i.e., design matrix) is used to define an ideal hemodynamic response function (HRF), which is convolved with the actual hemodynamic response and produces predictors of the BOLD response. For multiblock design, the temporal changes in the BOLD signal as predicted by the GLM conform to the “on-off” specifications set by the experimenter. Considering that the temporal profile of acupuncture is slow to develop and resolve, the magnitude of BOLD signal in rest period following the initial stimulation is unlikely to have returned to the initial (prestimulus) baseline level. Since fMRI analysis is an inherently contrastive methodology [32], the presence of activity during the baseline condition can seriously compromise the integrity of the sequential results. Due to the slow-acting agent of acupuncture, neural activities during rest periods may reduce, eliminate, or even reverse the sign of activities during stimulation conditions [31]. Therefore, the depiction of dynamic on-going acupuncture effects would be obtained in the absence of any assumption concerning the shape of the hemodynamic response, while the GLM's effectiveness in modeling such state-related activity is limited. In this line, a more flexible model, which captures consistencies in activation magnitude but allows for temporal variations, may be a more optimal choice instead.

**Figure 2:**
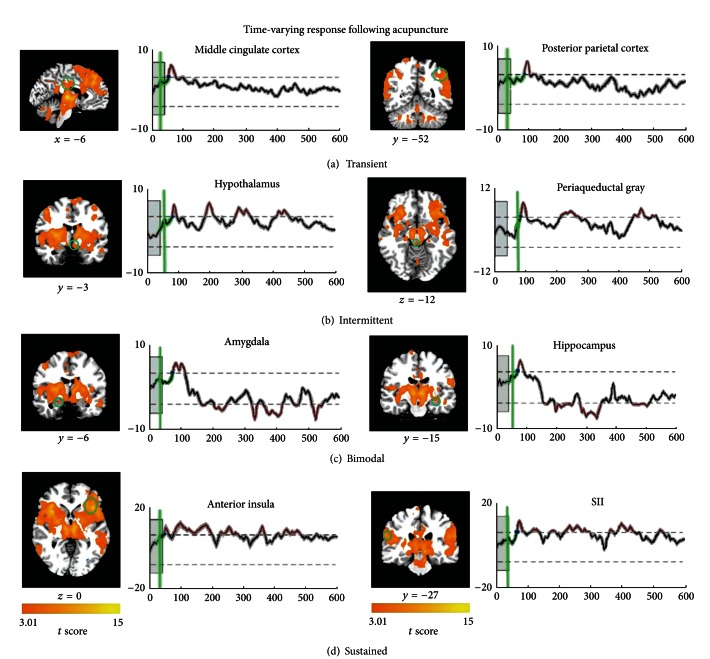
The baseline period was indicated by the shaded gray box, and the EWMA statistic was shown by the thick black line (corrected over time and FDR corrected at *α* = 0.05 over space), with gray shading denoting the standard error across participants. The estimated CP for onset activity was presented in green line. The control limits were shown by dashed lines. Abbreviations: SII: secondary somatosensory cortex (adapted from [33]).

Recently, our group has applied data-driven methods, such as the change-point approach with a hierarchical exponentially weighted moving average (HEWMA) analysis to model such slowly varying processes of acupuncture, of which the onset time and durations of underlying psychological activity were uncertain [33]. Our results demonstrated that BOLD signal changes induced by acupuncture shared a common feature that the identified voxels, containing a population of neurons, coded the temporal dimension (Figure 2). Notably, simply needling manipulation can evoke consistently increased signal changes in the wide pain-sensitive regions but more complex and time-varied neural responses during the poststimulus phrase. One possible explanation is that acupuncture manipulation, like kind of painful stimulus, generally involves a needling stimulation in deep tissue with both skin piercing and biochemical reactions to the tissue damage; this predominant experience may be primarily associated with excitatory responses in pain-related areas. As the effect of acupuncture may require a period of time to develop, its complex action on disassemble neural system may occur as time prolonged. For instance, the amygdala and perigenual anterior cingulate cortex (pACC) exhibited increased activities during the needling phrase while decreased gradually to reach significance below the baseline level. The periaqueductal gray (PAG) and hypothalamus presented saliently intermittent activations across the whole session. Relatively persistent activities were also identified in the anterior insula and prefrontal cortices. It is also noteworthy that there were remarkable overlapping brain regions involving acupuncture on both ST36 and nearby nonacupoint, the brain networks were more intrinsically heterogeneous and consisted of subsystems as time prolonged. Specifically, the sham networks consisted of a more time-independent subsystem that mainly included the sensorimotor and association cortices. In contrast, the acupuncture networks were much more extensive and time dependent, involving multiple neural circuitries. Previous investigations, focused on the spatial distribution of neural response to acute effects of acupuncture within a relatively short-term span, have argued that possible neural differences between the verum acupuncture and sham control are too subtle for detection in fMRI. As the effects of acupuncture may require a period of time to develop, the differences may only emerge over time when its delayed effect was being studied. To further test our hypothesis, we also adopted an electrophysiological imaging modality, namely, magnetoencephalography with a more sensitive temporal resolution on the order of milliseconds. Our findings showed that verum acupuncture can increase the connection degree between the temporal cortex (amygdala and hippocampus) and prefrontal cortex within delta (0.5–4 Hz) and beta (13–30 Hz) bands, while such effect occurred only in the delta band for sham control [34]. 

## 4. Interactions of Brain Network between Acupuncture and Expectancy

Acupuncture is a procedure in which fine needles are inserted into an individual at discrete points and then manipulated, with the intent of relieving pain. Clinical observations have shown that acupuncture analgesia is very effective in treating chronic pain, helping from 50% to 85% of patients (compared to morphine which helps only 30%). The analgesic effect of acupuncture is not a simple reflection or linear readout of incoming sensory information but can be substantially influenced by variations in individual physiological states. This inference can partially explain why the analgesic effect of acupuncture is generally characterized by tremendous interindividual variability. The existence of psychological factors in acupuncture analgesia when treating a patient's chronic pain is not unexpected, as with many medical treatments. There is still high skepticism whether acupuncture analgesia is predominately attributable to its physiological or just only psychological action. In the majority of randomized controlled trials (RCTs), it is generally concluded that acupuncture relevantly reduces pain but not more than a credible placebo procedure [35–37]. One recent systematic review using individual patient data meta-analyses to identify randomized controlled trials (RCTs) of acupuncture for chronic pain has promising results and indicates that acupuncture is effective for the treatment of chronic pain and is therefore a reasonable referral option. However, the difference of pain relief between acupuncture and placebo only obtain marginal significance. The substantial effect seen in the placebo acupuncture groups presents a significant challenge for both evaluating the efficacy and interpreting the effectiveness of acupuncture.

Distinct from the classical senses, pain is multifaceted and generally involves sensory, affective, and autonomic drive dimensions. The subjective evaluation of one's condition (e.g., “how do you feel?”) generally involves several complicated cognitive processes, which can be easily biased by previous experience and expectation [38]. In this regard, the availability of sophisticated brain imaging methods such as fMRI provides us objective techniques to enhance our understanding of the modulation mechanism of acupuncture. Recent studies have provided neuroimaging evidence to support different mechanisms underlying acupuncture in comparison with placebo/sham [39, 40]. Kong and colleagues examine both the interaction and dissociation between expectancy manipulation and acupuncture and demonstrate that expectancy could significantly influence acupuncture analgesia for the experimental pain, whereas acupuncture might specifically inhibit incoming noxious stimuli in comparison with the expectancy more involving the emotional circuit [39]. Harris et al. also investigate both short- and long-term effects of acupuncture versus sham treatment on in vivo *μ*-opioid receptor (MOR) binding availability in chronic fibromyalgia pain patients [40]. They suggest that acupuncture therapy could evoke both short-term and long-term increases in MOR binding potential in multiple pain and sensory processing regions associated with reductions in clinical pain, whereas such effect presented absent or even small reduction in the sham group. Another study enrolled fourteen patients with osteoarthritis of the thumb and designed two placebo controls (nonpenetrating Streitberger needle and an overt placebo) to explore both the specific effect of real acupuncture and the nonspecific effect of treatment expectation. The overt placebo was a nonpenetrating surface skin prick, bearing exactly the same stimulus as the Streitberger needle. However, patients were told in advance that this manipulation had no therapeutic effect. For the specific effect, by comparing the real acupuncture and Streitberger needle, the insula ipsilateral to the site of needling was activated to a greater extent during real acupuncture than during the placebo intervention. For the nonspecific effect, by comparing the Streitberger needle and overt placebo, the only difference was activation in dorsal lateral prefrontal cortex, rostral anterior cingulate cortex, and midbrain, suggesting a possible mechanism for the placebo effect involving acupuncture. The finding demonstrated that expectation in acupuncture treatment has a physiological effect on the brain network which mediates a potential nonspecific clinical response to acupuncture. These lines converge into one notion that divergent neural mechanisms may mediate specific dimensions of acupuncture effects in comparison with the placebo effects.

## 5. Deqi Sensations and Neural Responses by Acupuncture

In clinical settings, acupuncturists focused on “deqi” feeling during the needling treatment. This sensation was generally experienced by the patients and also by manipulating feeling of the acupuncturist when reaching the level of “qi” in the body. Deqi has recently drawn the attentions of many scientific researchers, and some studies propose that no appreciable therapeutic effect is obtained under a certain stimulation level, which is determined by the appearance of a particular sensation known as deqi [41–44]. One recent report investigated the characteristics of the “deqi” response in acupuncture at different acupoints (ST36, LI4, and LV3) and its association with distinct nerve fibers, compared with the conventional somatosensory or noxious stimuli. They indicated that aching, soreness, and pressure were most common sensations for different acupoints, followed by tingling, numbness, dull pain, heaviness, warmth, fullness, and coolness, and The sharp pain was regarded as inadvertent noxious stimulation. The most specific sensations of deqi were aching, soreness, pressure, and dull pain, in comparison of tactile stimulation control. Such complex composite of deqi sensations indicated involvement of nerve fibers at all levels (myelinated and unmyelinated nerve fibers). Particularly, the deeper muscle layers with its rich supply of slow conducting fibers may play the key role in acupuncture. It is consistent with the findings that deqi sensations are blocked after injection of procaine into the muscle beneath the acupoints. Following lumbar anesthesia, both deqi sensations and electromyography were completely abolished [45]. This phenomenon inferred that acupuncture-induced sensations were mainly generated from muscle, and the activity of polymodal-type receptors in deep tissues may play an important role [46]. This finding partly provides a clue to demonstrate the deqi with modern concepts in neurophysiology and bearing clinical relevance.

Given that deqi plays a pivot role in the therapeutic effect of acupuncture, it is noteworthy to find the relations between the brain activities and acupuncture-induced feelings. One study showed that acupuncture-induced deqi sensations without sharp pain primarily elicited widespread signal decreases in several brain areas, including the frontal pole, VMPF cortex, cingulated cortex, hypothalamus, reticular formation, and the cerebellar vermis, whereas sharp pain elicited signal increases in several areas, including the frontal pole and the anterior, middle and posterior cingulate [14]. They further inferred that acupuncture feeling without sharp pain are related to analgesia and antistress and deactivate the limbic-subcortical regions. By contrast, acupuncture feeling mixed with the sharp pain is associated with needling stimulation in deep tissue with skin piercing and biochemical reaction to tissue damage, and thus, the central effects of pain prevailed, exhibiting an integrated response with predominance of activation over deactivation in the cerebrocerebellar and limbic systems. Another research indicated that individual differences in the deqi scores can modulate the degree to which the right anterior insula was activated only following the verum acupuncture at ST36, compared with sham control [28]. The anterior insula has been widely accepted as a relay station integrating the centrally processed sensory information (visceral and autonomic) for its reciprocal connections with multiple brain regions [47]. This region, particularly the right anterior part, also plays a critical role in the interoceptive awareness of both stimulus-induced and stimulus-independent changes in the homeostatic state [48, 49], which enables us to regulate the organism's current state by initiating appropriate control signals toward the extrapersonal stimuli. This observation may suggest a key role of deqi in characterizing the central expression of acupuncture stimulation at ST36, which is relevant with its clinical efficacy in gastrointestinal analgesia.

## 6. Sham Control in Acupuncture Studies

Selecting the study design for both clinical and study investigations is prerequisite to answering the research question of interest whether acupuncture really works compared with control group. Thus, the central question is whether a particular acupoint needling manipulation performs better than another prescription, which is generally designed to determine an intervention's effectiveness compared to placebo in clinical trials. However, it is not known which aspects of the acupuncture treatment, such as the mode of stimulation or location of the acupuncture point, are specific to produce these physiological effects. The majority of neuroimaging researchers have suffered from selection of appropriate control challenges in order to address both the specific effect and relative effectiveness of acupuncture. There existed several control modalities. One approach is to apply the retractable nonpenetrating sham needle [50], which gives the impression of skin penetration without piercing the skin. As the needle is pushed against the skin, it causes a pricking sensation; but as increased pressure is applied, the shaft of the needle disappears into the handle, mimicking a “stage dagger.” The needle is held in position by a small adhesive plastic ring, which can also be used with the real needles to aid consistency and credibility. It mainly served as a control for nonspecific cognitive factors (e.g., expectation), whereas it may also lead to the subjects' bias toward the stimulation. To ensure the credibility of this control procedure, the research should enroll and pretest using acupuncture-naïve patients. Another control modality is the sham acupuncture, which is performed on a nearby nonacupoint with needle depth, stimulation intensity, and manipulation method all identical to those used in the real acupuncture. Although it is proved to be far from inactive and does in fact have a physiological effect [51–54], careful design and execution procedures can make it useful to assess the neural specificity of acupuncture with respect to different locations. Other control group included no treatment (or wait-list control), standard care, or combination of treatments. As for wait-list control, the research primarily attempts to factor out the natural history-induced effect. For standard care or another intervention, the study focuses more on the evaluation of the relative merits of different treatment interventions. Considering acupuncture is inherently multifaceted, the decision as to which control should be used will ultimately depend on the particular question that the research model plans to answer. It is thus important for researchers to clearly explain the target questions and select the appropriate control to match the purpose of the study. Using multiple controls at once is an optimal choice.

## 7. Further Considerations for Acupuncture Neuroimaging

Most of neuroimaging studies generally adopted the GLM analysis to focus on the spatial distribution of acupuncture-induced neural response to the acute effect of acupuncture. In fact, an acupuncture procedure typically involves two administration steps: (1) needling stimulation in deep tissue with skin piercing and biochemical reaction to tissue damage and (2) prolonged effects after the removal of acupuncture needle stimulation [28, 31]. In addition, evidence from both human behavior and animal studies indicates that a striking feature of acupuncture analgesia, in both human and animals, is its longevity—a delayed onset, gradual peaking, and gradual returning [29, 55, 56]. It is also substantiated that the physical needling stimulus, as well as the delayed effect of acupuncture, can similarly activate many areas of the brain [33]. Therefore, it is noteworthy to understand both acute and delayed effects of acupuncture on human brain. In addition, acupuncture, like many complex experiences, emerges from the flow and integration of information between specific brain areas. Great emphasis has been given to understand temporal interactions of these spatially defined brain regions, with consideration for how multiple levels of their dynamic activities in concert cause the processing of acupuncture.

 Due to the complexities of the therapeutic mechanisms of acupuncture, research into its neural mechanism has raised a number of difficult methodological issues. Variability in needling technique, deqi sensations, design paradigm, differences in neuroimaging hardware and software, and data postprocessing methods [57], may all account for many of the reported differences in brain response to acupuncture. Therefore, it is urgent to define a standardized reporting system to describe details of acupuncture manipulations [58]. Furthermore, specific selection criteria should also be used to ensure that a truly appropriate treatment is selected for the sham acupuncture treatment. The type of sham treatment chosen is dependent on the research question asked and the type of acupuncture treatment to be tested. For instance, using irrelevant acupoints can help to address the relative functional specificity of acupuncture. While acupuncture on same acupoints but with a different technique for the control condition can answer questions about the specific effects of those techniques, it is not known which aspects of the acupuncture treatment, such as the mode of stimulation or location of the acupuncture point, are specific to produce these physiological effects. In fact, a pilot study is needed to assist in determining if indeed it is an appropriate sham treatment and plays an important role in sample size calculations.

Previous acupuncture studies have generally adopted the multiblock design paradigm with repeated stimuli during a relatively short-term time span. Since acupuncture-related neural responses can be long lasting and not return to the baseline level immediately after the stimuli terminated, the “on-off” specifications set by block design may be violated; further analysis may be susceptible to errors of statistical significance [31]. In block designs, it is also difficult to disentangle the concurrent brain activity related to the needling manipulation from the brain activity associated with its sustained effect resulting from the same stimulation. Therefore, the nonrepeated event-related design paradigm may be more optimal in acupuncture studies. Furthermore, the model-based analysis (GLM) also becomes impractical when the precise timing and duration of acupuncture cannot be specified a priori. In other words, the depiction of dynamic on-going acupuncture effects would be obtained in the absence of any assumption concerning the shape of the hemodynamic response. For this purpose, the data-driven analysis, free of any hypothesis about the temporal profile of acupuncture-related changes, can be a more optimal choice instead.
